# Supplementary material for: Shared and Sex-Specific Genetic Risk for Parkinson’s Disease Risk Across European Populations
Source: medRxiv. 2026 Jan 2:2025.12.29.25343160. Preprint. [Version 1] doi: 10.64898/2025.12.29.25343160 (PMC12838296; doi:10.64898/2025.12.29.25343160)

medRxiv preprint doi: https://doi.org/10.64898/2025.12.29.25343160; this version posted January 2, 2026. The copyright holder for this preprint (which was not certified by peer review) is the author/funder, who has granted medRxiv a license to display the preprint in perpetuity.
It is made available under a CC-BY 4.0 International license .

Supplementary Figure 1: LocusCompare plot of the *GALC* locus (rs3213916).

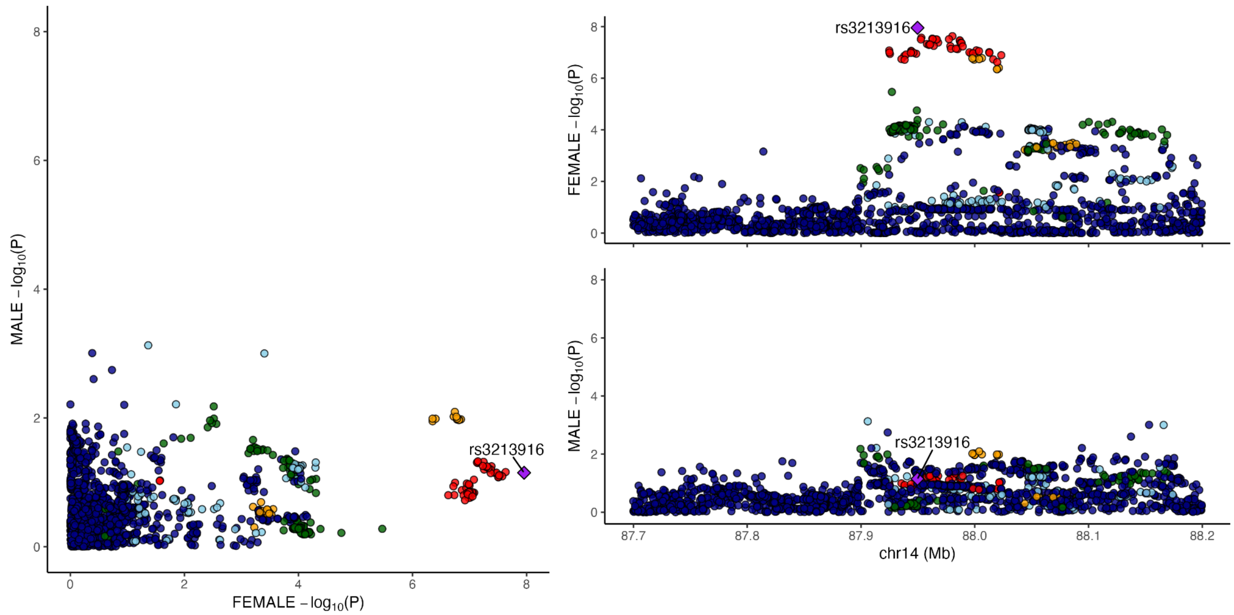

medRxiv preprint doi: https://doi.org/10.64898/2025.12.29.25343160; this version posted January 2, 2026. The copyright holder for this preprint (which was not certified by peer review) is the author/funder, who has granted medRxiv a license to display the preprint in perpetuity.
It is made available under a CC-BY 4.0 International license .

Supplementary Figure 2: LocusCompare plot of the *RBM8A* locus (rs71582802).

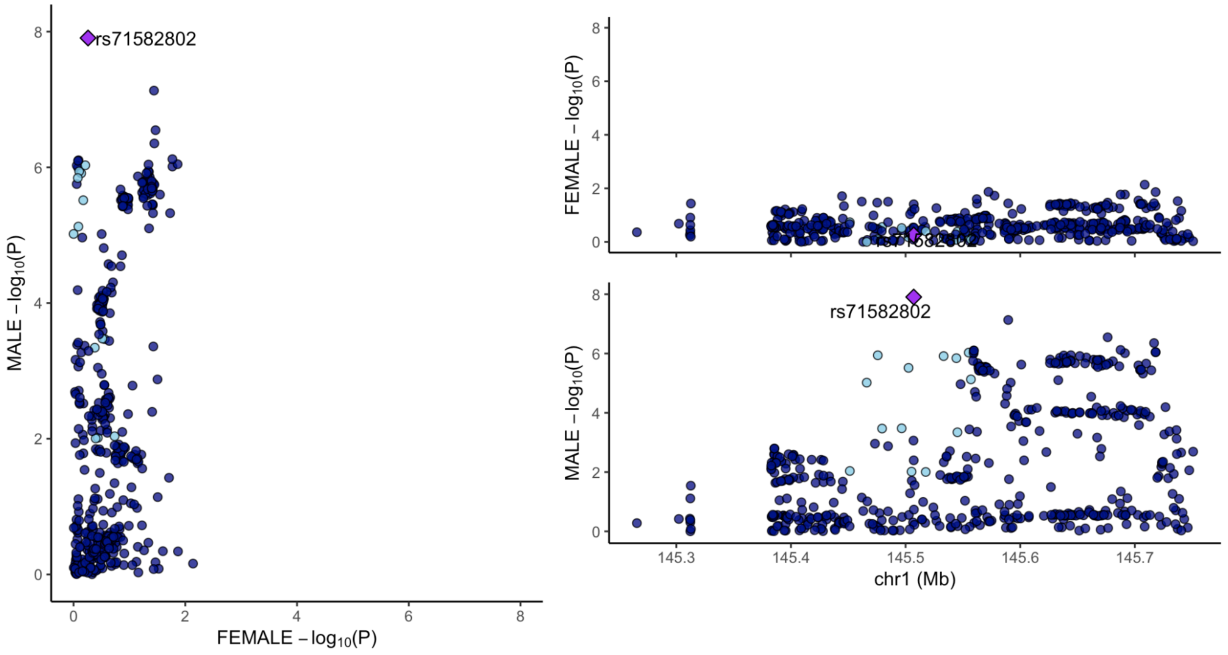

Supplementary Figure 3: LocusCompare plot of the *RERE* locus (rs301802).

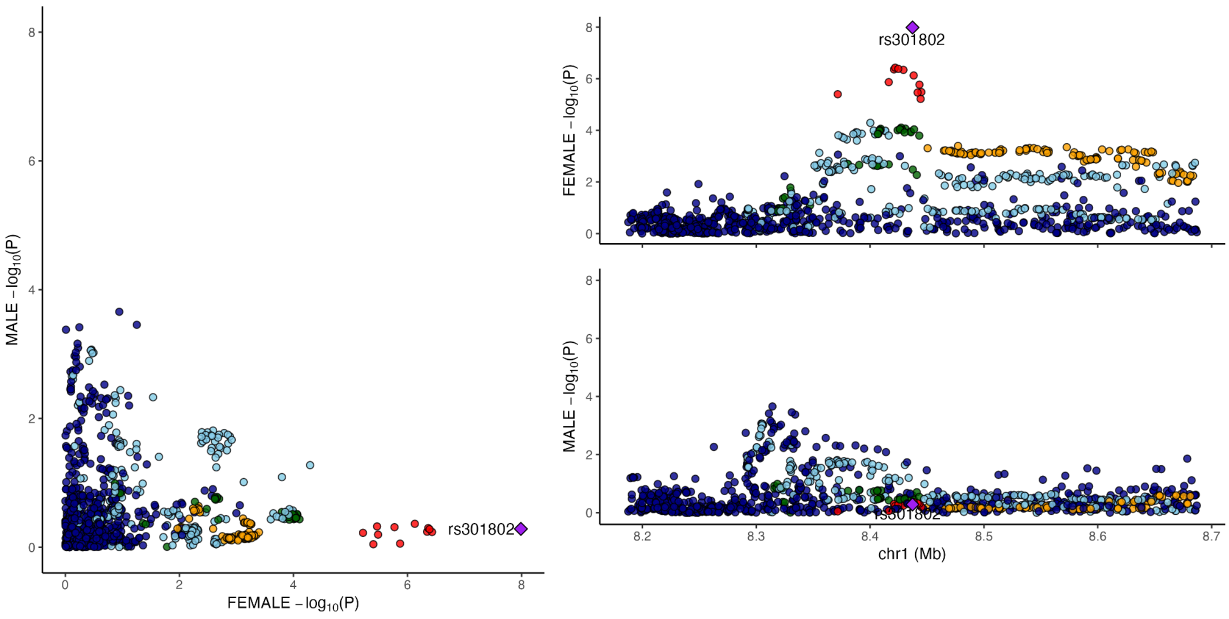

medRxiv preprint doi: https://doi.org/10.64898/2025.12.29.25343160; this version posted January 2, 2026. The copyright holder for this preprint (which was not certified by peer review) is the author/funder, who has granted medRxiv a license to display the preprint in perpetuity.
It is made available under a CC-BY 4.0 International license .

Supplementary Figure 4: LocusCompare plot of the *ARL6IP6* locus (rs7607599).

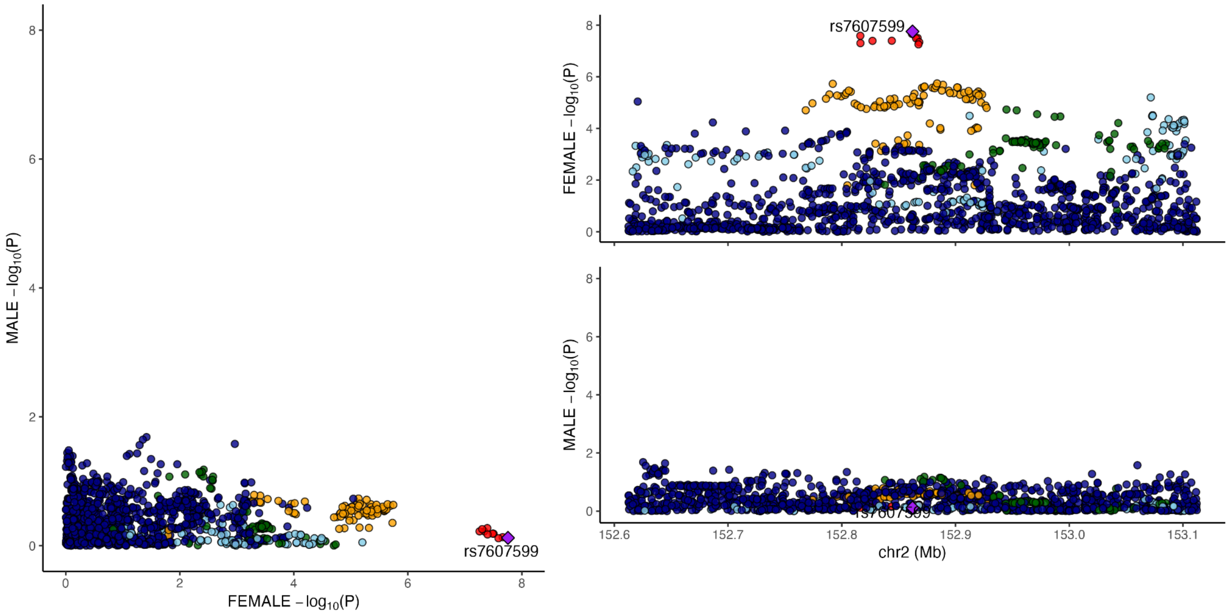

Supplement: Supplement 2 [file NIHPP2025.12.29.25343160v1-supplement-2.pdf]
